# High-altitude hypoxia and male reproductive dysfunction: testicular oxygen homeostasis, microenvironmental injury and spermatogonial niche vulnerability

**DOI:** 10.3389/fendo.2026.1939784

**Published:** 2026-09-03

**Authors:** Jiahao Li, Dongdong Meng, Bin Zhang, Changfeng Yang, Fulin Ma, Forong Li, Yanbao Wang, Zhaoyang Zheng, Zhanzhong Wang, Mengkun Li, Lingqi Liu, Dehui Chang

**Affiliations:** 1 School of Traditional Chinese and Western Medicine, Gansu University of Chinese Medicine, Lanzhou, China; 2 Department of Urology, The 940th Hospital of Joint Logistic Support Force of Chinese People's Liberation Army, Lanzhou, China; 3 Gansu Provincial Key Laboratory of Stem Cells and Gene Drugs, Lanzhou, China; 4 Northwest Minzu University, Lanzhou, China

**Keywords:** blood-testis barrier, high-altitude hypoxia, hypobaric hypoxia, male reproduction, reproductive endocrinology, spermatogonial stem cells, testicular microenvironment, testicular oxygen homeostasis

## Abstract

High-altitude hypoxia is a sustained environmental stressor that reduces inspired oxygen pressure and challenges tissue oxygen delivery. The male reproductive system is sensitive to oxygen availability, vascular perfusion, testosterone support, Sertoli-cell metabolic function and blood-testis barrier integrity. Human high-altitude exposure studies, mountaineering observations and animal hypobaric hypoxia models indicate that exposure to high altitude or hypobaric hypoxia is associated with reductions in sperm concentration, total sperm count, motility, viability and normal morphology, accompanied in experimental models by testicular structural injury, oxidative stress and reproductive endocrine alterations. However, the magnitude and reversibility of these changes vary substantially across studies. This variability suggests that altitude-related reproductive dysfunction is not a single linear consequence of hypoxia, but a dynamic process governed by exposure intensity, duration, acclimatization, systemic stressors and local testicular compensatory capacity. Building on the classical hypoxia-oxidative stress-germ-cell injury paradigm, this review proposes an integrated framework centered on testicular oxygen homeostasis, dual microenvironmental injury and spermatogonial niche vulnerability. In this model, high-altitude hypoxia first challenges the balance among oxygen delivery, microvascular blood flow, temperature and metabolism. When compensation fails, injury is propagated through two interconnected compartments: the interstitial vascular endothelial niche-Leydig-cell-immune axis and the seminiferous Sertoli-cell-blood-testis barrier-germ-cell support system. Peritubular myoid cells, the basement membrane and extracellular matrix form a boundary layer that links these compartments and participates in the spermatogonial stem-cell niche. The rete testis/efferent ductules, epididymal segmental microenvironment, blood-epididymis barrier and seminal extracellular vesicles are best interpreted as downstream or modifying layers rather than primary mechanisms. Whether injury reaches spermatogonia or the spermatogonial stem-cell niche may determine whether recovery is rapid and complete or delayed and incomplete. The review also identifies evidence gaps, including direct measurement of local testicular oxygen tension, compartment-specific experimental designs, niche-targeted endpoints and longitudinal high-altitude/lowland return cohorts.

## 1 Introduction

High-altitude hypoxia is a persistent and biologically important environmental stressor at high altitude, arising primarily from reduced barometric pressure and the consequent decrease in inspired oxygen partial pressure. Increasing military deployment, construction and mining activities, tourism, mountaineering and migration to high-altitude regions have made male reproductive health during altitude exposure an important topic at the intersection of reproductive medicine, environmental medicine and high-altitude physiology. Existing studies indicate that exposure to high altitude or hypobaric hypoxia can alter semen quality, testicular histology, reproductive hormone secretion and germ-cell fate (1–5). These observations support the clinical relevance of altitude exposure, particularly for men with fertility plans or repeated occupational exposure. In this context, hypobaric hypoxia (HH) represents the principal oxygen-related component of terrestrial high-altitude exposure and is therefore widely used experimentally to simulate altitude-associated hypoxic stress. However, experimental HH does not reproduce the entire high-altitude environment, where hypoxia may coexist with cold exposure, physical exertion, sleep disruption and energy imbalance. Normobaric hypoxia, which reduces oxygen availability without lowering barometric pressure, provides additional mechanistic information but is not physiologically identical to HH. Accordingly, real-altitude observations and HH models constitute the primary evidence base of this review, whereas normobaric and other hypoxia models are used mainly to support mechanistic interpretation.

Earlier explanations often emphasized a simple sequence of hypoxia, oxidative stress, germ-cell apoptosis and reduced semen quality (2, 3, 6). Although this pathway accounts for many downstream findings, it does not adequately explain three important observations. First, systemic hypoxia does not necessarily imply immediate pathological hypoxia within the testis; acute reductions in inspired oxygen can be partly compensated by increases in testicular blood flow and oxygen extraction (7). Second, Leydig-cell responses to hypoxia are exposure-mode dependent: acute or intermittent hypoxia may trigger adaptive responses, whereas sustained or severe hypoxia is more likely to suppress steroidogenesis (8–11). Third, semen parameters, testosterone levels and testicular histology do not necessarily recover at the same rate after return to low altitude (12, 13).

This review therefore reframes high-altitude hypoxia-induced male reproductive dysfunction through an integrated model of testicular oxygen homeostasis, dual microenvironmental injury and spermatogonial niche vulnerability. In this framework, high-altitude hypoxia first challenges the local balance among oxygen delivery, microvascular perfusion, temperature and metabolism. When compensation is exceeded, injury is propagated through the interstitial vascular endothelial niche-Leydig-cell-immune axis and the seminiferous Sertoli-cell-blood-testis barrier (BTB)-germ-cell support system. Peritubular myoid cells, the basement membrane and extracellular matrix (ECM) connect these compartments as a structural and signaling boundary layer. The critical determinant of recovery is whether injury remains limited to mature sperm or late germ cells, or whether it extends to spermatogonia and the spermatogonial stem cell (SSC) niche. To clarify the terminology used throughout this framework, the working definitions, conceptual status and supporting evidence for the principal terms are summarized in **Table 1**.

**Table 1:** Working definitions, conceptual status and evidence basis of core concepts used in this review.

Concept	Working definition	Status in this review	Supporting references
Testicular oxygen homeostasis	Dynamic balance among oxygen delivery, local blood flow, temperature and metabolism; the gate between adaptation and decompensation under high-altitude hypoxia.	Integrative working concept	The physiological basis is supported by established evidence on testicular oxygen delivery, compensatory blood flow and oxygen extraction, as well as the importance of temperature and metabolic homeostasis (1, 7, 14). The interpretation of oxygen homeostasis as an upstream “gate” between compensation and decompensation is proposed in this review.
Interstitial microenvironment	Compartment composed of vascular endothelial cells, microvessels, Leydig cells, macrophages, peritubular structures and paracrine factors; regulates oxygen delivery, steroidogenesis, immune homeostasis and SSC-niche signaling.	Literature-based compartment, operationally defined here	The individual cellular interactions and functions are supported by studies of Leydig-cell biology, testicular endothelial cells, macrophage–Leydig interactions, immune privilege, and interstitial regulation of the SSC niche (15–21).
Seminiferous microenvironment	Compartment composed of Sertoli cells, the BTB and germ cells; maintains metabolic support, immune separation and spermatogenic differentiation.	Literature-based compartment	Sertoli-cell metabolic support and germ-cell energy dependence are well established, as are the structural and regulatory functions of the BTB (22–26). The term is used here to organize these established relationships within the dual-microenvironment framework.
Boundary layer	Peritubular myoid cells, basement membrane and ECM; a structural and signaling interface linking interstitial signals to the basal SSC niche.	Author-defined integrative construct	The anatomical components and the contributions of peritubular and interstitial cells to SSC maintenance are supported by existing literature (21, 27–30). However, the term “boundary layer” and its interpretation as a continuous interface linking the two compartments are introduced in this review as an organizing concept rather than an established anatomical designation.
Spermatogonial niche	Microenvironment formed by SSCs/spermatogonia together with Sertoli cells, peritubular cells, endothelial cells, Leydig cells, macrophages and local growth factors that maintain spermatogenic reserve.	Established biological concept; extended here to the interpretation of injury reversibility	The cellular and signaling basis of the spermatogonial/SSC niche is supported by extensive experimental evidence (16, 18, 27–32). In this review, extension of hypoxic injury to spermatogonia or the SSC niche is proposed as a potential determinant of delayed or incomplete reproductive recovery; direct altitude-specific evidence for this interpretation remains limited.
Post-testicular microenvironment	Rete testis/efferent ductules, epididymal segmental microenvironment, blood-epididymis barrier, seminal metabolites and extracellular vesicles; influences sperm transport, concentration, maturation, storage and early functional manifestations.	Integrative umbrella term used in this review	The functions of the efferent ductules, epididymal environment, blood-epididymis barrier, and extracellular vesicles are supported by established reproductive-biology literature (33–41). Evidence directly linking these components to high-altitude hypoxia remains limited; therefore, they are treated here primarily as downstream or modifying layers rather than established primary mechanisms of altitude-related reproductive injury.

## 2 Search strategy and literature synthesis

This narrative review was prepared through a structured literature search and mechanism-oriented synthesis. Literature searches were updated to June 2026. PubMed/MEDLINE and Web of Science were used as the main bibliographic databases, with Google Scholar used as a supplementary source for identifying additional relevant studies and tracing references. PubMed Central and publisher websites were used primarily for full-text retrieval and reference checking. The core search combined altitude/hypoxia-related terms (“high altitude” OR “high-altitude hypoxia” OR “hypobaric hypoxia” OR “normobaric hypoxia” OR “simulated altitude”) with male reproductive terms (testis OR spermatogenesis OR sperm OR “male fertility” OR “Leydig cell” OR “Sertoli cell” OR “blood-testis barrier” OR “spermatogonial stem cell”), with additional mechanism-specific terms added when appropriate.

The literature was synthesized according to its relevance and biological proximity to the review question. Human high-altitude observations, mountaineering or trekking cohorts, occupational exposure studies and animal hypobaric hypoxia models were used to define the altitude-related reproductive changes and the most relevant tissue-level findings. Cell-based studies, normobaric-hypoxia studies and general reproductive-biology literature were used selectively to strengthen mechanisms already aligned with the main framework, including Leydig-cell steroidogenesis, Sertoli-cell metabolic support, BTB dynamics, regulated cell death and SSC-niche signaling. Mechanisms derived mainly from non-altitude models are therefore presented as complementary explanations or testable hypotheses, not as replacements for high-altitude or hypobaric hypoxia observations. Studies were included when they provided direct evidence of male reproductive changes after high-altitude or hypoxic exposure, or when they addressed mechanisms directly relevant to the framework of this review. Studies without a clear relationship to male reproductive function or to the mechanisms discussed here were excluded.

Retrieved publications were screened first by title and abstract, followed by full-text assessment when necessary to determine relevance. During evidence synthesis, study quality was considered qualitatively according to study design, sample size, appropriateness of the exposure model and controls, consistency of outcome measurements, and potential confounding factors. Greater interpretive weight was given to human longitudinal studies and well-controlled experimental studies, whereas findings from small cohorts, heterogeneous exposure models or indirect mechanistic studies were interpreted more cautiously.

HH and normobaric hypoxia both reduce oxygen availability, but they are not physiologically identical. Differences in ventilatory response, fluid balance, nitric-oxide biology and acute mountain sickness-related responses mean that real-altitude or hypobaric evidence should remain the primary context when discussing high-altitude male reproductive effects (42).

## 3 Direct evidence linking high-altitude or hypobaric hypoxia exposure to male reproductive impairment

Available evidence relevant to male reproductive responses to high-altitude or hypobaric hypoxia encompasses longitudinal and prospective human studies, cross-sectional and other observational investigations, controlled animal experiments, and mechanistic laboratory studies. These evidence types differ substantially in their proximity to clinically relevant reproductive outcomes and should therefore not be interpreted as equivalent. Human studies directly assessing semen parameters provide the closest evidence for reproductive changes, whereas endocrine or sexual-function studies provide supportive evidence and animal or molecular studies primarily contribute tissue-level and mechanistic information. **Table 2** organizes the available studies accordingly and summarizes their major study characteristics, exposure and follow-up, reproductive assessment methods, principal findings, and study-specific strengths and limitations; closely related studies with similar evidence roles are summarized jointly where appropriate to reduce redundancy.

**Table 2:** Human, animal and mechanistic evidence relevant to male reproductive responses to high-altitude or hypobaric hypoxia.

Study	Study characteristics and exposure	Reproductive assessment and main findings	Study-specific strengths and limitations
A. Longitudinal/prospective human studies with direct semen or reproductive outcomes			
He et al. (12)	52 healthy Han Chinese male soldiers; 18-21y; ~1400 to 5380 m for 12 mo; Assessment/follow-up: Baseline; 6 and 12 mo at altitude; 6 mo after return.	WHO 2010; semen after 4–7 d abstinence; CASA; PRL/LH/FSH/testosterone. Semen count/concentration, motility and viability worsened during exposure and largely recovered after descent.	Strength: Large repeated-measures baseline-exposure-recovery cohort. Limitations: No concurrent control; homogeneous young military cohort; no pregnancy/live-birth outcomes.
Okumura et al. (43)	3 male climbers; age 20–37y; >5100 m for 21–24 d and >6700 m for 4–5 d; Assessment/follow-up: Pre; 1 mo, 3 mo and 2 y post-expedition.	Repeated semen and hormone testing. Sperm count fell at 1/3 mo, morphology worsened transiently and testosterone declined; recovery occurred later.	Strength: Very long (2-y) recovery follow-up. Limitations: n=3; no control; expedition-related workload/cold/sleep/energy confounding; semen protocol details limited.
Pelliccione et al. (44)	7 trained male mountaineers; age 33 ± 9 y; ~5900 m, 22 d of high-altitude exercise; Assessment/follow-up: Pre; hormones during exposure; semen/hormones 10 d after return.	Conventional semen analysis, CASA, sperm DNA fragmentation by flow cytometry and hormones. Sperm concentration decreased; DNA fragmentation did not clearly worsen.	Strength: Prospective semen + DNA-integrity + endocrine assessment. Limitations: Small sample; no unexposed comparator; exercise/weight loss occurred with hypoxia.
Verratti et al. (45)	Small male trekking cohort (secondary evidence: n=7); age 47 ± 8y; ~900 to 5895 m over 5 d; Assessment/follow-up: Pre/post expedition.	Conventional semen parameters and reproductive hormones. Forward motility decreased; most other semen variables were unchanged; LH increased.	Strength: Direct pre/post semen and endocrine assessment. Limitations: Very short exposure; small cohort; intense exercise; no non-altitude control; participant details incompletely reported.
Verratti et al. (38)	5 healthy Italian male lowlanders; age 44 ± 15 y (25–63); 300-km Himalayan trek over 19 d; Assessment/follow-up: Pre; 10 d and 70 d post-expedition.	WHO semen analysis after 3–4 d abstinence plus seminal redox/lipid profiling. Sperm concentration/volume fell after trekking with redox/metabolic changes.	Strength: Repeated recovery sampling with detailed biochemical endpoints. Limitations: n=5; wide age range; no control; trekking, diet and other environmental stressors confound hypoxia.
Zheng et al. (46)	Healthy young lowlanders; control (n=31) plus 1-mo (n=4) and 3-mo (n=45) exposure groups; age 18–24 y; Lhasa 3600 m vs Chongqing ~400 m; Follow-up: none after return.	Semen volume, pH, concentration and sperm morphology by light microscopy. Concentration decreased after 3 mo; head-deformity distribution changed.	Strength: Low-altitude comparator and exposure-duration contrast. Limitations: Independent groups, not repeated measures; no recovery assessment;very small sample size in the 1-month group (n=4).
B. Cross-sectional, observational and supportive human studies			
Gu et al. (47)	2798 men: 1111 native Tibetans vs 1687 Han men on plains; median age 30 y (16–40); long-term residence comparison; Design: cross-sectional.	WHO semen after 2–7 d abstinence; morphology/motility; hormones in 474 and DFI in 526. Several motility, morphology, endocrine and DFI measures differed, but sperm output was not uniformly lower.	Strength: Largest human sample; multiple reproductive endpoints. Limitations: Cross-sectional; ancestry, adaptation, lifestyle and environmental differences limit causal attribution.
Human endocrine studies (48–50)	21 men, 49.5 ± 11.8 y, serial 550–7050 m exposure (48); 26 men, 18–35 y, 4300 m for 3 wk with energy-balance comparison (49); 7 eugonadal men, 3500 m with days 1/7/18 and 7-d post-return sampling (50).	FSH/LH/testosterone, PRL and broader endocrine/metabolic profiling; no semen assessment. Altitude-associated gonadal-axis changes were reported, with partial adaptation/recovery and modulation by energy balance.	Strengths: Repeated endocrine measurements; included a controlled energy-balance comparison (49). Limitations: Small/selected cohorts; no semen or direct testicular endpoints; endocrine inference is supportive rather than direct.
Other supportive human studies (51–53)	338 Chilean miners, 40.22 ± 6.61 y, chronic intermittent HH (51); 447 men in Ali, 31.24 ± 7.52 y, cross-sectional (52); one 37-y climber during a 43-d expedition to ~5800 m (53).	PSA/testosterone/SaO2/hemoglobin (51); libido, sleep and mental-health questionnaires (52); repeated RigiScan measurements (53). These studies extend evidence to prostate-related, libido and erectile outcomes; none assessed semen.	Strengths (51, 52): Large real-world cross-sectional samples in and repeated objective erectile measurement in. Limitations: Heterogeneous indirect endpoints, substantial environmental/behavioral confounding, and a single-case design in (53).
Wang et al. (54)	119 analyzed men with varicocele; mean age 24.8 y; mean altitude 4442 m/residence 25.1 mo; semen subgroup n=49 (Lhasa 20, Ali 29); Design/follow-up: Retrospective; no post-exposure follow-up.	Semen volume, viability, graded motility, viscosity, liquefaction and WBCs. Several indices were poorer at ~4500 vs ~3650 m.	Strength: Direct semen comparison between two plateau locations. Limitations: All subjects had varicocele; retrospective clinical design; no healthy low-altitude control.
C. Controlled animal studies with reproductive and testicular outcomes			
Saxena (13)	7 Male rhesus monkeys; age NR; simulated 4411 m for 21 d; Assessment/follow-up: Early recovery to ~3 wk post-exposure.	Semen volume, sperm count/concentration, motility, pH/fructose and testicular histology. Semen quality declined with epithelial degeneration/spermatogenic arrest; early recovery was partial.	Strength: Primate model with semen plus direct testicular histology. Limitations: Older study; animal n/age incompletely reported; limited recovery information.
Farias et al. (55, 56)	24 male Wistar rats, 342 ± 14 g, n=8/group (55), continuous/intermittent HH at ~4600 m for 60 d; 36 male Wistar rats, 10 wk, 225 ± 14 g, n=6/group, IHH 96 h/96 h for 32 d with ascorbate intervention (56).	Testicular histology/seminiferous epithelium, round-spermatid metabolism, epididymal sperm-related endpoints and oxidative markers. Sustained/intermittent HH produced structural and oxidative spermatogenic injury; ascorbate modified selected responses.	Strengths: Defined chamber exposures with complementary histological/redox endpoints. Limitations: Different exposure durations; included antioxidant intervention (56); standardized fertility endpoints were limited.
Liao et al. (57)	Adult male Wistar rats; control plus 5000-m HH for 5, 15 or 30 d; group n/age/weight not fully reported in accessible methods.	Histology/electron microscopy, germ-cell DNA content by flow cytometry and TUNEL apoptosis; longer HH also reduced epididymal sperm. Injury was duration-dependent.	Strength: Clear exposure-duration gradient with complementary assays. Limitations: Incomplete animal characteristics in accessible methods; no recovery phase.
Zepeda et al. and Yao et al. (58, 59)	30 male SD rats, 10 wk, n=5/group, intermittent HH over 32 d with blueberry extract (58); 24 male SD rats, 12 wk, n=8/group, IHH 96 h/96 h x4 over 32 d with pentoxifylline; assessment 7 d later (59).	Both assessed testicular histology/morphometry, apoptosis and oxidative injury; additionally measured caudal sperm count/density and motility (59). IHH caused structural/redox injury and reduced sperm quality; interventions attenuated selected abnormalities.	Strengths: Controlled IHH models with tissue/redox endpoints; included direct sperm assessment (59). Limitations: Small groups and intervention-focused designs; extrapolation to unmodified human exposure is limited.
Bai et al. (60)	32 prepubertal male Wistar rats; simulated 5000 m HH for 3 wk; Assessment/follow-up: 3-wk normobaric recovery; later sperm production at postnatal day 63.	Testis/body weight, testosterone, histology, spermatogenic/Sertoli/Leydig cells, apoptosis and later sperm production. Some spermatogenic deficits persisted after recovery.	Strength: Exposure-recovery design with later spermatogenic output. Limitations: Prepubertal model differs substantially from typical adult human exposure.
D. Mechanistic and omics animal studies			
Omics and cell-resolved studies (61–63).	male mouse HH model, simulated 5500 m for 35 d (61); 8-wk B6-129S1-background males, >6/group, HH for 1–4 wk with normoxic recovery (62); 6-wk C57BL/6 males, n=10/group, 5000 m for 4 wk, n=3/group for scRNA-seq (63).	sperm phenotype + LC-MS/MS RNA-modification profiling (61); histology, sperm abnormalities, spermatogonia/SSC function and transcriptomics/alternative splicing (62); histology/IF + scRNA-seq (63). Together they identify RNA-regulatory, spermatogenic and cell-specific responses, with partial recovery in (62).	Strengths: Complementary molecular approaches provide pathway- and cell-specific resolution; includes time-course/recovery and functional SSC assessment (62). Limitations: Heterogeneous mouse models; omics associations require functional validation; scRNA-seq n=3/group; no direct human evidence.
Chai et al. (64)	Male rat HH model + Leydig-cell experiments; animal age/n NR; simulated 5000 m; Follow-up: not reported.	Sperm count/motility/malformation, testosterone, Leydig-cell number and ferroptosis markers. Findings support Leydig-cell ferroptosis as a candidate pathway.	Strength: Combines whole-animal phenotype with targeted mechanism assays. Limitations: Animal/cell evidence; incomplete animal characteristics; relative importance of ferroptosis in humans is unknown.

NR, not reported or not verifiable in the accessible primary source; CASA, computer-assisted sperm analysis; DFI, sperm DNA fragmentation index; GPX4, glutathione peroxidase 4; IHH, intermittent hypobaric hypoxia; LC-MS/MS, liquid chromatography-tandem mass spectrometry; MDA, malondialdehyde; PRL, prolactin; PSA, prostate-specific antigen; SaO2, arterial oxygen saturation; scRNA-seq, single-cell RNA sequencing; TUNEL, terminal deoxynucleotidyl transferase dUTP nick-end labeling.

Human studies provide the clinical anchor for altitude-related male reproductive impairment, but they should be interpreted with attention to exposure duration, ascent profile, workload, sleep, nutrition, abstinence interval and sampling practice. The longitudinal study at 5380 m remains particularly informative because it followed young men through prolonged exposure and lowland return, showing that sperm count, concentration, motility, viability and reproductive hormones can change during altitude exposure and that many semen indices may recover after descent, while testosterone may recover on a different time course (12). Expedition and trekking studies add shorter-term observations in which concentration, forward motility, morphology, seminal redox balance and seminal metabolic profiles may change (38, 43–46). Endocrine and sexual-function studies further indicate that high-altitude reproductive effects may involve hormonal-axis regulation, libido and erectile physiology, but these outcomes are particularly sensitive to sleep, fatigue, psychological stress and energy balance (48–50, 52, 53). Together, these studies support altitude-associated reproductive vulnerability without implying universal or irreversible infertility.

The human literature also shows why high-altitude exposure cannot be reduced to oxygen pressure alone. Mountaineering, trekking and occupational settings often combine hypoxia with exercise load, cold exposure, dehydration, sleep disruption, psychological stress and negative energy balance. Long-term high-altitude residents and plateau clinical populations require separate interpretation because adaptation, ancestry, lifestyle, selection bias and coexisting male-factor conditions may modify semen or endocrine outcomes (47, 54). Occupational chronic intermittent HH studies broaden the relevance of the topic for miners and other high-altitude workers, but prostate/PSA endpoints and workplace confounding should not be overread as direct semen-mechanism evidence (51). These factors do not weaken the relevance of hypoxia; rather, they require careful interpretation of testosterone, semen and recovery data within the broader high-altitude environment.

**Figure 1:**
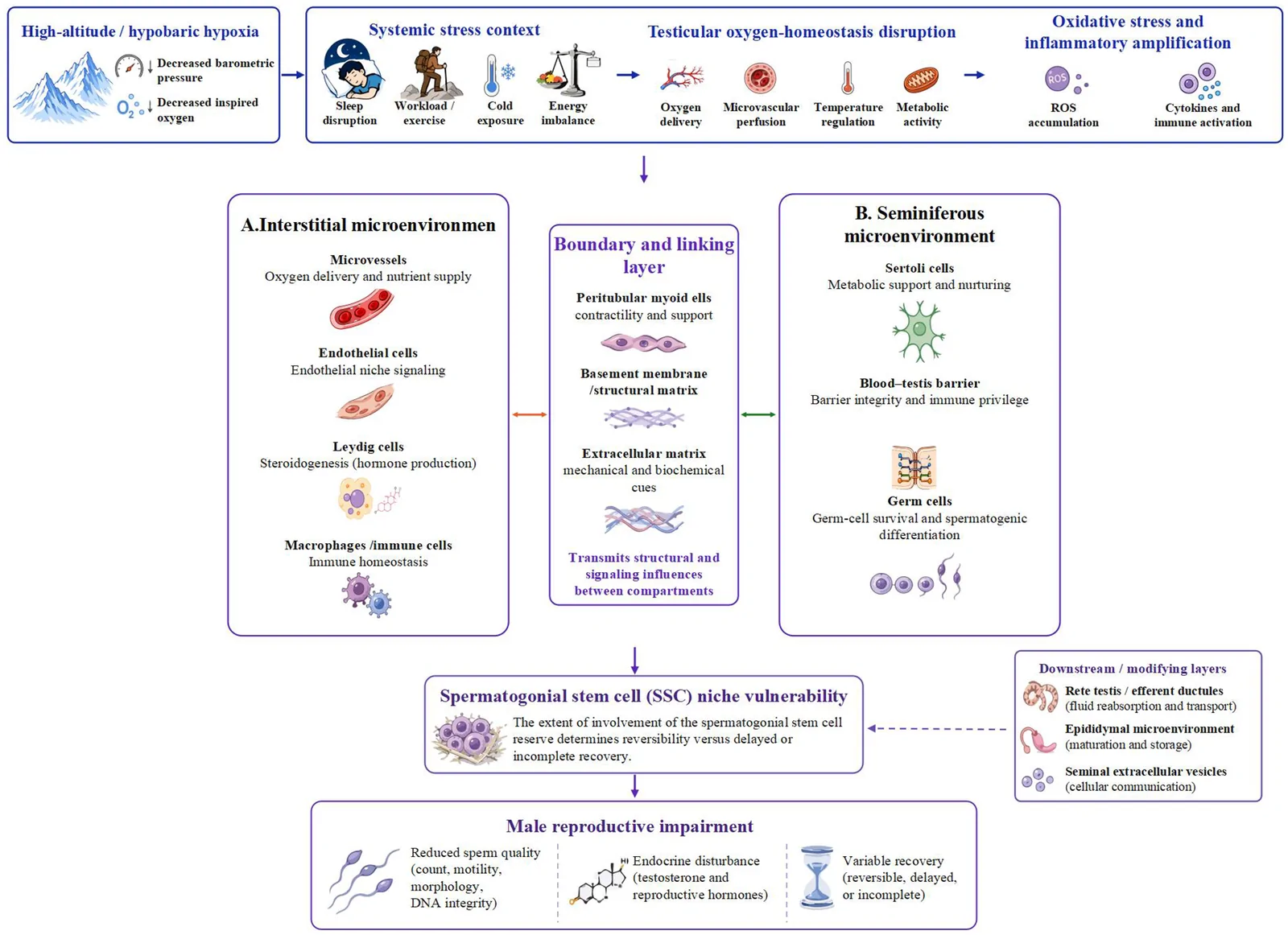
Conceptual framework of high-altitude hypobaric hypoxia-induced male reproductive dysfunction. Failure of testicular oxygen homeostasis propagates injury through interacting testicular microenvironments and downstream modifiers, shaping reproductive impairment and recovery.

Animal hypobaric hypoxia models provide the histological and mechanistic bridge that human cohorts cannot fully supply. Intermittent simulated altitude exposure in rhesus monkeys reduced semen volume, sperm count and motility, and induced seminiferous epithelial degeneration and spermatogenic arrest with partial reversibility (13). Rodent HH models repeatedly show reduced testicular mass or seminiferous epithelial height, germ-cell apoptosis, oxidative stress and impaired epididymal sperm quality after sustained or intermittent exposure (55–60). Single-cell, transcriptomic and RNA-modification analyses further suggest that peritubular myoid cells, germ-cell populations and spermatogenic regulatory pathways may be sensitive to hypobaric hypoxia (61–63). These findings support an association between high-altitude or hypobaric hypoxia exposure and semen/testicular impairment, while still requiring caution regarding model heterogeneity, exposure intensity and cross-species generalizability. Taken together, the human and experimental evidence suggests that altitude-related reproductive impairment is better understood as a multilevel process involving testicular oxygen homeostasis, local microenvironmental injury and the preservation of spermatogenic capacity. This overall framework is summarized in **Figure 1** and provides the basis for the mechanistic sections that follow.

## 4 Testicular oxygen homeostasis: from physiological hypoxia to decompensation

The testis is not a uniformly high-oxygen organ. Relatively low local oxygen tension may limit excessive oxidative reactions, yet spermatogenesis, steroidogenesis and seminiferous epithelial maintenance still require adequate oxygen and metabolic substrates (1). Accordingly, high-altitude exposure should not be equated with immediate pathological testicular hypoxia. Reduced inspired oxygen pressure may be partly compensated by increased ventilation, erythropoiesis, blood-flow redistribution and tissue oxygen extraction. Locally, microvascular regulation and metabolic redistribution may delay injury.

**Figure 2:**
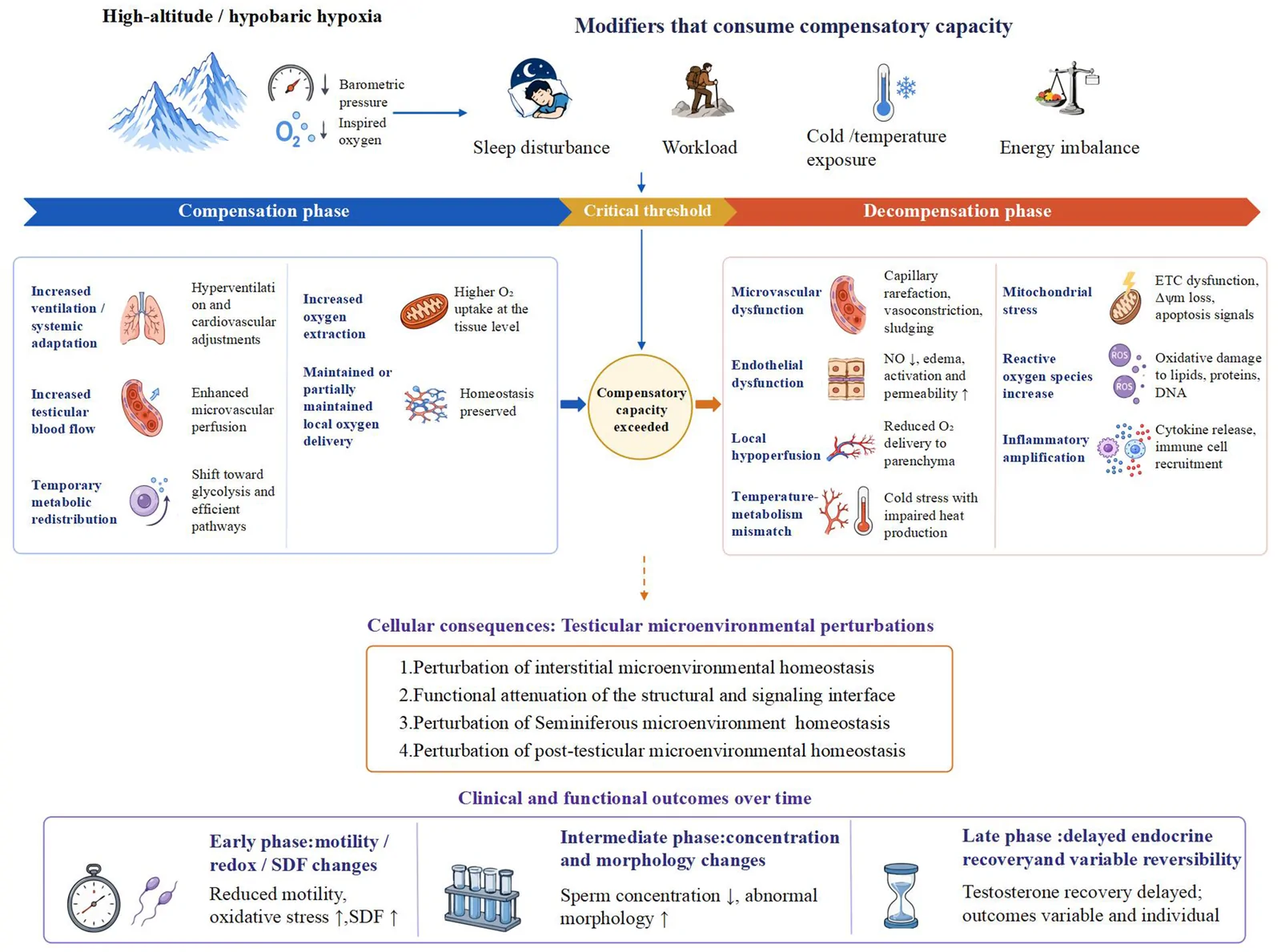
Compensation-to-decompensation transition of testicular oxygen homeostasis. Early compensatory responses may preserve oxygen delivery, whereas prolonged or intensified systemic stress can exhaust this reserve and promote microenvironmental dysfunction and reproductive injury.

A key physiological study showed that reduced oxygen content in inspired air increased testicular blood flow and oxygen extraction, helping maintain testicular oxygen delivery without immediate evidence of anaerobic metabolism (7). This finding indicates that reduced inspired oxygen does not necessarily cause immediate testicular hypoxia. A more accurate model is that the testis has a limited compensatory capacity; prolonged, severe or repeated hypoxia can exceed this threshold and shift local oxygen homeostasis from adaptation to decompensation. This transition from initially effective compensation to progressive decompensation, together with the systemic factors that may accelerate loss of compensatory capacity, is illustrated in **Figure 2**.

Testicular oxygen homeostasis is closely linked to thermal and metabolic homeostasis. Hypoxia-induced increases in blood flow may support oxygen delivery but can also alter heat exchange and metabolic demand. Cold exposure, insulation, exercise and sympathetic activation at high altitude may further affect scrotal and testicular temperature regulation. Evidence from heat-stress studies shows that elevated testicular temperature can impair spermatogenesis, sperm DNA integrity and semen quality (14). Thus, altitude-related testicular stress is better described as a disturbance of oxygen delivery, blood flow, temperature and metabolism rather than oxygen deficiency alone.

Possible decompensation features include microvascular endothelial dysfunction, altered vascular remodeling, inadequate oxygen extraction, local heat imbalance, mitochondrial dysfunction and sustained hypoxia-inducible factor (HIF) signaling. Because Leydig cells are located close to interstitial microvessels, small changes in oxygen delivery may first affect steroidogenesis. By contrast, seminiferous tubules lack direct vascular penetration and rely heavily on Sertoli-cell transport and metabolic support. Testicular oxygen homeostasis is therefore the upstream gate linking systemic altitude exposure to dual microenvironmental injury.

## 5 Dual microenvironmental mechanisms and connecting layers

**Figure 3:**
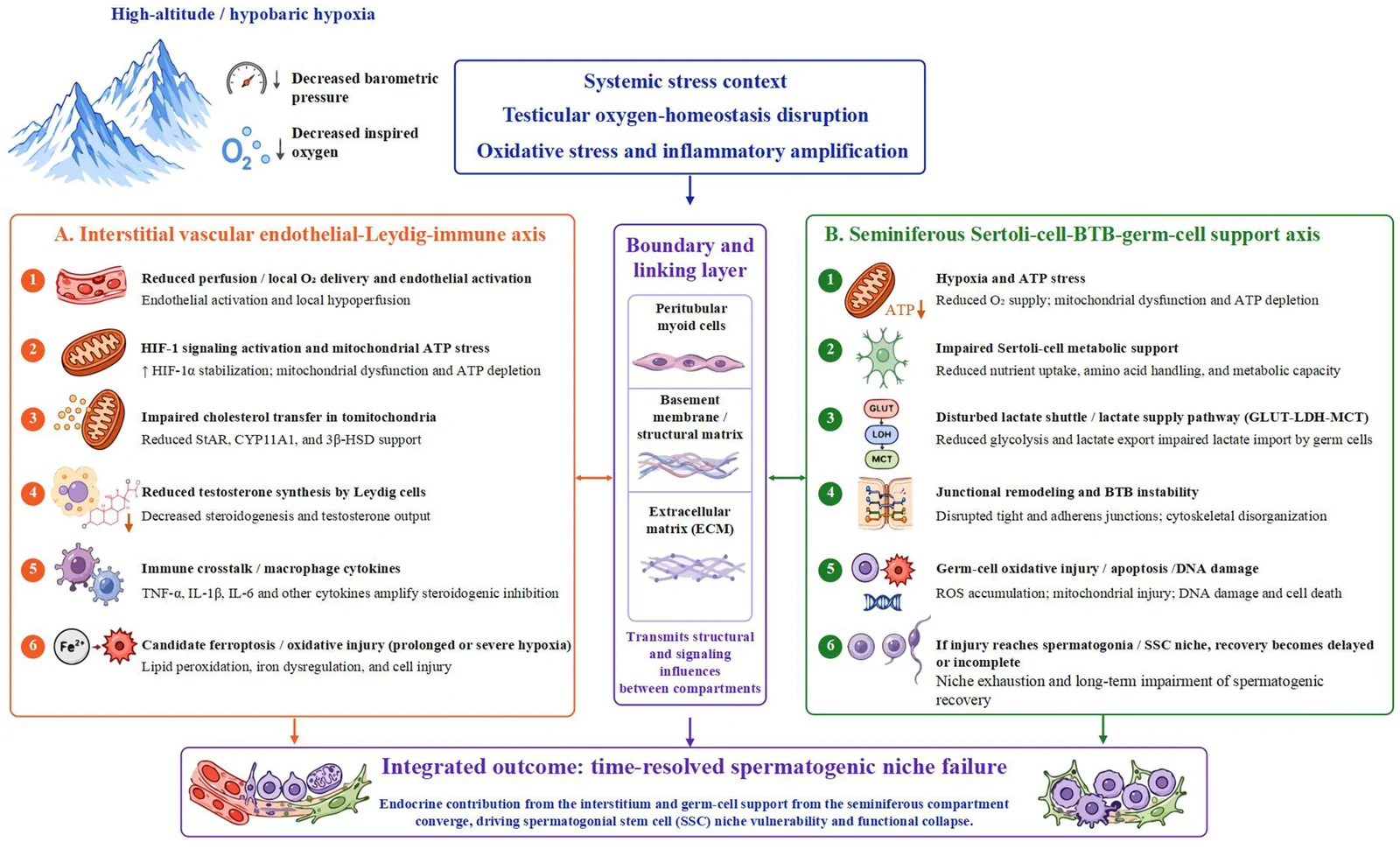
Dual-microenvironment model under high-altitude hypobaric hypoxia. Disrupted testicular oxygen homeostasis affects the interstitial endothelial–Leydig–immune axis and the seminiferous Sertoli–BTB–germ-cell system across the peritubular/ECM boundary.

Once the compensatory capacity of testicular oxygen homeostasis is exceeded, reproductive injury is unlikely to remain confined to a single cell type or pathway. Instead, the anatomical and functional organization of the testis suggests two closely interacting sites of vulnerability: the interstitial compartment, where vascular endothelial, Leydig-cell and immune signals converge, and the seminiferous compartment, where Sertoli-cell metabolic support, BTB integrity and germ-cell differentiation are coordinated. These two compartments are anatomically distinct but functionally connected through peritubular, basement-membrane and ECM interfaces. This dual-microenvironment organization is summarized in **Figure 3** and provides the structure for the following subsections.

### 5.1 Interstitial microenvironment: vascular endothelial niche, Leydig cells and immune paracrine signaling

Leydig cells in the interstitial compartment are responsible for testosterone synthesis and secretion, and their location near microvessels, macrophages and peritubular structures makes them both targets and mediators of hypoxic stress. Steroidogenesis requires coordinated luteinizing hormone (LH)-dependent cyclic adenosine monophosphate/protein kinase A signaling, steroidogenic acute regulatory protein (StAR)-mediated mitochondrial cholesterol transfer, mitochondrial oxidative metabolism and sequential activity of CYP11A1, 3β-hydroxysteroid dehydrogenase, CYP17A1 and 17β-hydroxysteroid dehydrogenase (15, 65). Therefore, altitude-related interstitial injury should be considered as a disturbance of vascular oxygen delivery, mitochondrial cholesterol handling, steroidogenic enzyme activity and paracrine regulation rather than as a simple fall in circulating testosterone.

Testicular microvessels are not merely conduits for oxygen and nutrients. Testicular endothelial cells are also components of the germline stem-cell niche and can support long-term spermatogonial stem cell (SSC) maintenance through paracrine factors such as glial cell line-derived neurotrophic factor (GDNF) (16). Microvascular dysfunction could therefore influence reproduction through two connected routes: impaired oxygen delivery to interstitial Leydig cells and altered endothelial niche signaling to spermatogonia. This link is important because it connects the upstream oxygen-homeostasis model to both endocrine output and spermatogenic reserve.

The effects of hypoxia on Leydig-cell steroidogenesis appear biphasic and exposure-mode dependent. Sustained or severe hypoxia tends to suppress testosterone synthesis, whereas acute or intermittent hypoxia may transiently preserve steroidogenic activity through vascular endothelial growth factor signaling, autophagy or lipophagy (8–11, 66). At the molecular level, HIF-dependent suppression may occur at the mitochondrial entry step of steroidogenesis: reduced NRF1-dependent StAR expression and hypoxia-inducible factor 1 (HIF-1)-mediated repression of StAR transcription may limit cholesterol transfer into mitochondria, thereby reducing CYP11A1-dependent pregnenolone formation and downstream androgen synthesis (9, 11, 15). Mitochondrial reactive oxygen species (ROS), membrane-potential loss and ATP depletion may further aggravate this bottleneck during prolonged exposure. Thus, the most defensible interpretation remains a compensation-decompensation process rather than a unidirectional inhibition model.

Ferroptosis and other regulated cell-death pathways refine this Leydig/interstitial module when hypoxia becomes prolonged or severe. A simulated 5000 m model reported lipid peroxidation, iron accumulation, downregulation of the cystine/glutamate antiporter SLC7A11/xCT–glutathione peroxidase 4 (GPX4) axis, ROS accumulation and reduced Leydig-cell proliferative activity (64). These findings support a hypoxia-lipid peroxidation-steroidogenic injury pathway (67, 68), while apoptosis markers such as Bax/Bcl-2 imbalance and caspase activation are more closely related to irreversible cell loss (69, 70). Autophagy, lipophagy and mitophagy may initially protect Leydig cells by maintaining organelle quality or lipid-substrate availability, whereas persistent p62 accumulation, abnormal LC3-II turnover or impaired mitophagic clearance may indicate failure of this protective flux (10, 71–74). These pathways should complement rather than replace the steroidogenic, mitochondrial and oxidative-stress frameworks.

The testicular immune microenvironment may amplify injury between interstitial and tubular compartments. Under physiological conditions, testicular macrophages, Sertoli cells, Leydig cells and peritubular cells contribute to immune privilege and paracrine homeostasis; macrophages also interact with Leydig cells, vasculature and the spermatogonial niche (17–21). Hypoxia, ROS, lipid-peroxidation products and damage-associated molecular patterns released from damaged germ cells may promote macrophage activation and inflammatory cytokine release. Tumor necrosis factor-α, interleukin-1β and interleukin-6 can further suppress steroidogenesis, weaken Sertoli-cell metabolic support and disturb BTB dynamics (75, 76). Inflammation should therefore be viewed not merely as a downstream consequence, but as a potential amplifier of the transition from compensation to decompensation.

### 5.2 Seminiferous microenvironment: sertoli-cell support, BTB dynamics and germ-cell vulnerability

Sertoli cells form the functional core of the seminiferous microenvironment. They provide structural support, nutrients and paracrine signals for developing germ cells and build the BTB through specialized junctional complexes (22–24, 77–79). Because blood vessels do not directly enter seminiferous tubules, meiotic and post-meiotic germ cells depend strongly on Sertoli-cell transport, glycolytic buffering and barrier-mediated control of the tubular milieu.

Sertoli cells display active glycolytic metabolism and convert glucose into lactate, which is exported to germ cells through monocarboxylate transport systems. Lactate is not only an energy substrate but also participates in germ-cell survival, meiosis and differentiation (22, 23, 78–83). This pathway involves glucose transporter-mediated glucose entry, lactate dehydrogenase-dependent pyruvate-to-lactate conversion and monocarboxylate transporter family-mediated lactate export. Under high-altitude or HH stress, compensatory glycolysis may initially support adluminal germ cells, but ATP shortage, mitochondrial-glycolytic imbalance or defective lactate export could convert Sertoli-cell adaptation into insufficient germ-cell support. Direct evidence that high-altitude hypoxia specifically downregulates MCT4 remains limited, so this remains a testable pathway-level hypothesis.

The BTB consists of tight junctions, basal ectoplasmic specializations, desmosome-like junctions and gap junctions. Claudin-11, occludin, ZO-1, N-cadherin, β-catenin, connexin 43 and F-actin contribute to its structure and dynamic remodeling (24–26, 77, 84–86). Hypoxia-related ROS, inflammatory cytokines, ATP shortage and cytoskeletal stress may disturb junctional protein localization, phosphorylation, endocytosis, recycling, cytoskeletal anchoring and resealing. Therefore, BTB impairment is better interpreted as active junctional remodeling than passive leakage. Future high-altitude or HH studies should examine junctional localization, F-actin organization, ultrastructure and permeability assays, rather than relying only on total junctional-protein abundance.

### 5.3 Peritubular myoid cells, basement membrane and ECM: a boundary layer linking two compartments

Peritubular myoid cells, the basement membrane and ECM form a structural and signaling interface between interstitial and seminiferous compartments. Peritubular myoid cells drive seminiferous tubule contractions and sperm transport toward the rete testis and, together with Sertoli cells, vascular cells and macrophages, contribute to the SSC niche through cell-type-specific signals, including peritubular-cell-derived GDNF and colony-stimulating factor 1, Sertoli-cell-derived CXCL12 and insulin-like growth factor 1-associated signaling (21, 27, 28, 87–90).

This boundary layer provides a structural and signaling basis for viewing the two compartments as a continuous niche. Changes in interstitial oxygen delivery, steroidogenic activity and immune signaling may influence Sertoli-cell support and basal spermatogonial localization across the peritubular/basement membrane/ECM interface. Single-cell work suggests that peritubular myoid cells may be among hypoxia-sensitive populations (63), but the effects of high-altitude hypoxia on peritubular contractility, basement-membrane permeability and ECM remodeling remain insufficiently characterized. Taken together, the evidence discussed above defines the relatively well-supported core of the proposed framework, although the strength of altitude-specific evidence differs among individual mechanisms. The supporting literature, defensible conclusions and major limitations of these core mechanistic modules are summarized in **Table 3**.

**Table 3:** Core mechanistic modules, supporting literature and recommended interpretation.

Mechanistic module	Supporting literature	Reasonable conclusion	Limitations
Semen/testicular impairment after altitude or HH	Human altitude studies, mountaineering observations and HH animal models (12, 13, 38, 43–46, 55–60).	Altitude/HH exposure is associated with semen quality decline and experimental testicular injury.	Do not state that all exposed men develop irreversible infertility.
Leydig-cell steroidogenic disruption	Hypoxia cell/animal work; HIF-1 and NRF1-StAR axes (9, 11).	Sustained or severe hypoxia can suppress steroidogenesis.	Short-term/intermittent hypoxia may induce compensation; mode dependence is essential.
Vascular endothelial niche	Testicular endothelial-cell niche studies (16).	Microvessels may support SSCs through paracrine signals as well as oxygen/nutrient delivery.	Direct altitude effects on endothelial niche signaling remain to be validated.
Immune microenvironment	Macrophage-Leydig and macrophage-SSC niche studies (17, 18).	Inflammation may amplify the transition from compensation to decompensation.	Separate homeostatic immune support from pathological inflammation.
Sertoli-cell lactate support	Sertoli metabolism and germ-cell energy studies (22, 23, 78–83).	Sertoli lactate supply is essential for spermatogenesis.	Altitude-specific disruption of individual transporters remains a hypothesis.

HIF-1, hypoxia-inducible factor 1; NRF1, nuclear respiratory factor 1; StAR, steroidogenic acute regulatory protein.

## 6 Spermatogonial and SSC niche vulnerability as a determinant of reversibility

Declines in semen quality after high-altitude exposure do not necessarily imply permanent infertility. The central question for recovery is whether the spermatogenic reserve is preserved. Spermatogonia and SSCs are the cellular foundation of long-term spermatogenesis. Their self-renewal and differentiation depend on a niche composed of Sertoli cells, the basement membrane, peritubular myoid cells, endothelial cells, Leydig cells, macrophages and local growth factors (16, 18, 21, 27–31, 91).

Semen parameters reflect downstream reproductive output at a given time, whereas testosterone production, Sertoli-cell support, BTB dynamics and the SSC niche represent upstream regulatory and spermatogenic capacity. If hypoxia mainly affects mature sperm or late spermatogenic cells, removal from altitude may allow recovery through the retained spermatogonial pool. If injury extends to SSC self-renewal, spermatogonial proliferation or niche signaling, restoration of normoxia may not immediately restore sperm output and recovery may be delayed or incomplete.

Recent evidence links hypoxia to impaired spermatogonial proliferation through a Septin2-PP2A/AKT-related pathway (32). Hypoxia has been reported to reduce Septin2 transcription, destabilize the PP2A/B56γ complex, attenuate AKT phosphorylation and induce G1/S arrest in spermatogonia. This mechanism is valuable because it links cytoskeletal organization, cell-cycle entry and niche-dependent proliferation, moving the mechanistic focus upstream from terminal sperm injury to the spermatogenic reserve. However, it remains a developing mechanism that requires validation in altitude-dose, hypobaric hypoxia and recovery designs.

Additional germ-cell mechanisms may extend the same niche-vulnerability framework. Chronic hypoxia has been linked to disruption of the round-to-elongated spermatid transition through an ASXL2-EZH2-CEP162/TUBB3-related microtubule pathway, suggesting a possible route from sustained hypoxic stress to impaired spermatid elongation, flagellar construction and later sperm structural quality (92). Hypoxia-related RNA modifications and post-transcriptional regulation, including Am, Gm, m^7^G, m²_2_G and m^5^C-related marks and associated regulatory proteins, have also been reported in mouse testis, sperm and germ-cell models (61, 62, 93). These mechanisms help complete the germ-cell injury layer, but they should be used to guide endpoint selection and mechanistic hypotheses rather than treated as established biomarkers in exposed men.

## 7 Post-testicular downstream layers and systemic modifiers

After spermatogenesis, sperm traverse the straight tubules, rete testis and efferent ductules before entering the epididymis. The rete testis-efferent ductule system regulates luminal fluid reabsorption, ionic composition, osmolarity and ciliary movement, thereby contributing to sperm concentration and transport. Efferent ductules are not passive conduits; they have essential fluid-reabsorptive functions relevant to male fertility (33). Direct evidence that high-altitude hypoxia affects this region is lacking, but downstream transport and fluid-handling abnormalities are plausible contributors to early motility changes before sperm concentration changes become evident.

The epididymis is essential for acquisition of progressive motility, membrane stabilization and fertilization competence. Its segmental microenvironment, the blood-epididymis barrier, epithelial secretion, luminal pH and ion composition, antioxidant systems and local inflammatory status all influence sperm maturation and storage (34, 35). Short-term exposure to high altitude may initially reduce progressive sperm motility before any evident change in sperm concentration occurs, and may also be accompanied by disturbances in seminal redox balance and alterations in lipid metabolite composition (36, 38, 45). Therefore, the epididymal segmental microenvironment and blood-epididymis barrier are reasonable downstream layers, but they should not be presented as proven primary mechanisms of high-altitude male reproductive impairment.

Seminal extracellular vesicles (EVs) may provide another post-testicular layer. Epididymosomes and prostasomes transfer proteins, lipids, RNAs and regulatory molecules to sperm and participate in membrane remodeling, motility acquisition, capacitation, acrosome reaction and immune protection (39–41). Whether high-altitude hypoxia alters seminal EV cargo or function remains unknown. This topic is best positioned as a future research direction that may help explain seminal metabolic changes and early sperm functional impairment.

Oxidative stress remains the most consistently supported shared mechanism across altitude and hypoxia models. Hypoxia can impair mitochondrial electron transport, increase ROS production, deplete antioxidant capacity and enhance lipid peroxidation, thereby damaging sperm membranes, DNA and mitochondria and affecting steroidogenesis and Sertoli-cell junctional function (37, 56, 58, 75, 94–97). Inflammation, autophagy imbalance and ferroptosis should be integrated into the dual-microenvironment model as stress modules rather than listed as isolated mechanisms.

Two emerging mechanisms deserve cautious placement. First, hypoxia-sensitive epididymal cells may promote NK-cell activation via the KLF4-ASH1L-ICAM-1 axis and thereby contribute to epididymal epithelial and blood-epididymis barrier injury (98). Second, high-altitude-associated microbial changes and gut-derived succinate accumulation may amplify testicular macrophage inflammation through immune-metabolic signaling (99). These mechanisms may help explain individual variability and systemic amplification, but their clinical relevance and therapeutic implications remain unproven. These and other candidate or downstream mechanisms, together with their current evidence and limitations, are summarized in **Table 4**.

**Table 4:** Candidate and downstream mechanistic modules, supporting literature and recommended interpretation.

Mechanistic module	Supporting literature	Reasonable conclusion	Limitations
BTB dynamics	BTB structure and signaling studies (24–26, 77, 84–86).	BTB integrity is central to seminiferous microenvironment stability.	Direct functional evidence under high-altitude hypoxia is limited.
Ferroptosis	Leydig-cell hypoxia ferroptosis and male reproductive ferroptosis reviews (64, 67, 68).	May contribute to lipid-peroxidation injury during prolonged/severe hypoxia.	Should not replace oxidative stress and steroidogenesis as main frameworks.
SSC niche	SSC niche biology and hypoxia-Septin2 evidence (16, 18, 29–32, 91).	Spermatogonial/SSC niche involvement may determine recovery potential.	More high-altitude models and human data are needed.
Post-testicular microenvironment	Efferent ductule, epididymal, blood-epididymis barrier and extracellular-vesicle literature (33–36, 39, 40).	May explain early motility decline and seminal metabolic changes.	Direct altitude evidence is limited; EVs should not be treated as a primary mechanism.
Remote immune-metabolic amplification	High-altitude gut-derived succinate evidence (99).	A candidate explanation for individual variability and inflammatory amplification.	Does not justify routine clinical microbiome or receptor-targeted interventions.
Autophagic-flux imbalance	Hypoxia/autophagy and Leydig adaptive-response studies (10).	Autophagy, lipophagy and mitophagy may be initially protective, but persistent flux failure may aggravate mitochondrial and steroidogenic injury.	Altitude-specific direction, timing and cell specificity require validation.
Spermiogenic cytoskeletal remodeling	Chronic-hypoxia spermatid microtubule pathway evidence (92).	May link sustained hypoxia to spermatid elongation, flagellar construction and sperm structural quality.	Candidate chronic-hypoxia mechanism; direct high-altitude/human evidence is limited.
RNA modification and post-transcriptional regulation	Hypoxia-related testis/sperm RNA-modification studies and transcriptome regulation of testicular tissues (61, 62).	May affect mRNA stability, translation and sperm RNA cargo during spermatogenic stress.	Useful for omics endpoints, but not yet an established clinical biomarker.

EVs, extracellular vesicles.

## 8 High-altitude adaptation and translational considerations

The effect of high-altitude hypoxia on the testis should not be described as uniformly injurious. Short-duration or mild hypoxic exposure may induce increased blood flow, enhanced oxygen extraction, metabolic remodeling, autophagy and endocrine compensation. Injury becomes more likely when exposure duration and intensity increase, or when hypoxia is combined with cold, intense exercise, inflammation, sleep disruption and insufficient energy intake. *In vitro* hypoxia transcriptomics in yak Sertoli cells suggests remodeling of glucose metabolism, PI3K-AKT/MAPK/RAS, junctional, immune, autophagy and apoptosis pathways (96). Because reproductive responses and subsequent recovery are time dependent, clinical assessment should also distinguish the pre-exposure, exposure and post-return phases. A practical framework for assessment and follow-up across these stages is summarized in **Table 5**.

**Table 5:** Suggested assessment and follow-up for men exposed to high altitude.

Stage	Suggested assessment	Interpretation
Pre-exposure/baseline	Fertility history, prior semen analysis, baseline semen parameters and TMSC; SDF and hormones when fertility planning is important.	Establishes personal baseline and reduces misinterpretation of single results.
During exposure	Record altitude, duration, sleep, exercise, nutrition, cold, medication and symptoms; hormones if clinically indicated.	Separates hypoxia effects from systemic stressors.
1 month after return	Repeat motility, viability, SDF, seminal redox or inflammatory markers; exploratory EV markers in research settings.	Captures mature sperm, epididymal/seminal and early functional recovery.
Approximately 3 months after return	Repeat concentration, total count, TMSC, morphology and relevant hormones.	Corresponds more closely to a complete spermatogenic cycle.
6 months after return	Refer to andrology/reproductive medicine if abnormalities persist; consider genetic, endocrine, imaging and ART pathways.	Helps identify delayed recovery or persistent endocrine/niche-related impairment.

ART, assisted reproductive technology; EVs, extracellular vesicles; SDF, sperm DNA fragmentation; TMSC, total motile sperm count.

The hypothalamic-pituitary-gonadal (HPG) axis provides an important systemic endocrine link between whole-body adaptation to high-altitude exposure and local testicular function. Endocrine studies show that adrenal, thyroid and gonadal axes can change with altitude, with partial recovery during adaptation but persistent disruption at higher altitudes (48, 100, 101). Within this broader endocrine response, human studies have reported altitude-associated changes in LH, follicle-stimulating hormone (FSH) and testosterone, although the direction and magnitude of these responses vary with exposure duration, acclimatization and energy balance (12, 48–50). These findings suggest that reproductive endocrine responses to altitude are dynamic rather than uniformly suppressive. Changes in gonadotropin drive may influence Leydig-cell steroidogenic activity and Sertoli-cell support, thereby modifying the capacity of the local testicular microenvironment to adapt to hypoxic and metabolic stress. Conversely, impaired testicular steroidogenesis may alter gonadal feedback and further modify systemic endocrine regulation. The HPG axis can therefore be viewed as a systemic regulatory node linking whole-body responses to altitude with local reproductive function.

Real high-altitude exposure is also accompanied by sleep fragmentation, periodic nocturnal hypoxia, cold exposure, increased physical load, appetite loss and negative energy balance, all of which may further influence HPG-axis activity and reproductive hormone profiles. Accordingly, reduced testosterone or sexual symptoms after altitude exposure should not automatically be interpreted as either primary central suppression of the HPG axis or isolated Leydig-cell failure. Most available studies assess circulating LH, FSH and testosterone rather than hypothalamic or pituitary function directly; endocrine findings should therefore be interpreted together with gonadotropin levels, sampling time, exposure duration, sleep, exercise and nutritional status.

Potential clinical groups include military and occupational personnel, miners and construction workers, tourists and mountaineers, long-term migrants to high altitude and men planning conception around altitude exposure. Semen assessment should follow standardized procedures such as the World Health Organization (WHO) sixth edition laboratory manual (102) and should be interpreted in the broader context of male-infertility evaluation principles (103, 104). A single abnormal semen analysis after altitude exposure should not be used to diagnose persistent infertility. A 1-month reassessment may capture early motility, viability, DNA fragmentation or redox recovery; approximately 3 months better corresponds to a complete spermatogenic cycle; a 6-month follow-up can help identify delayed recovery or persistent endocrine or niche-related impairment.

## 9 Evidence gaps and future research priorities

Several major knowledge gaps remain across basic, translational and clinical research. At the basic level, the local testicular oxygen threshold for decompensation, compartment-specific causal pathways, SSC-niche involvement and post-testicular contributions remain unresolved. Translationally, it is unclear which experimental mechanisms or biomarkers track human reproductive injury and recovery. Clinically, the natural history, risk thresholds and effective interventions are insufficiently defined.

First, direct physiological evidence on local testicular oxygen regulation under reduced inspired oxygen remains limited. Increased testicular blood flow and oxygen extraction can preserve testicular oxygen delivery during reduced inspired oxygen exposure (7), but the local testicular oxygen threshold at which physiological compensation becomes decompensation remains undefined. Controlled animal studies should therefore use graded hypobaric-hypoxia intensity and duration with predefined exposure-recovery time points, integrating local oxygen tension, blood flow, temperature and metabolic readouts.

Second, the relative causal contributions of the interstitial vascular endothelial niche-Leydig-immune axis and the seminiferous Sertoli-BTB-germ-cell axis remain unclear. Compartment- or cell-specific perturbation studies with temporal sampling are needed to determine which compartment is affected first and whether injury in one compartment drives dysfunction in the other.

Third, existing HH evidence indicates that hypoxia can alter undifferentiated spermatogonial populations while SSC regenerative capacity may remain preserved in experimental models. However, whether HH directly damages spermatogonia/SSCs or their supporting niche, and whether niche injury predicts incomplete recovery, remain unresolved. Exposure-recovery studies should combine SSC/niche markers with functional measures of spermatogonial reserve and later sperm production rather than relying on mature sperm endpoints alone.

Mechanistic measurements should remain hypothesis-driven and should not substitute for appropriate study design. Representative pathway-linked markers and functional endpoints are summarized in **Table 6** and should be selected according to the specific biological question.

**Table 6:** Unresolved evidence gaps and priorities for basic, translational and clinical research.

Unresolved knowledge gap	Research priority/question	Recommended study design	Key measurements/endpoints
A. Basic research			
Local testicular oxygen thresholds and the timing of compensation-to-decompensation are undefined.	Define dose- and time-dependent changes in local testicular oxygen homeostasis.	Controlled animal HH studies with graded altitude/duration and exposure-recovery sampling.	Local oxygen tension and blood flow; local temperature; HIF-1α; ATP/lactate; representative endothelial markers (e.g., CD31/VE-cadherin); testicular histology and spermatogenic output.
The relative causal roles of interstitial and seminiferous compartments are unclear.	Determine temporal order and cross-compartment causality.	Compartment- or cell-specific perturbation studies with matched time-course controls.	Leydig steroidogenic function (e.g., StAR/CYP11A1); endothelial and inflammatory activity; Sertoli metabolic flux; BTB permeability/junctional localization; germ-cell survival. Where relevant, representative stress markers may include SLC7A11-GPX4 and LC3-II/p62.
Whether SSC/niche injury determines delayed or incomplete recovery is unknown.	Test whether niche integrity predicts spermatogenic recovery.	Exposure-recovery animal studies with functional SSC/niche assessment.	Spermatogonial/SSC abundance and regenerative capacity; representative markers (e.g., PLZF/GFRα1, ID4/UCHL1); niche signaling (GDNF/CXCL12-AKT); later sperm production after recovery.
The independent contribution of post-testicular transport and maturation defects is uncertain.	Separate testicular production defects from ductal/epididymal maturation defects.	Segment-resolved animal studies across testis, rete/efferent ductules and epididymal regions.	Fluid reabsorption and ciliary function; blood-epididymis barrier permeability; luminal pH/ion composition; redox/immune status; representative extracellular-vesicle cargo; CASA-based sperm motility and maturation indices.
B. Translational research			
Experimental mechanisms and biomarkers have not been robustly linked to human reproductive injury or recovery.	Identify markers that track semen impairment and recovery across experimental and human studies.	Parallel animal-human studies using harmonized exposure descriptors, sampling windows and reproductive endpoints.	Standardized semen parameters, TMSC and SDF; LH/FSH/testosterone; selected mechanistically anchored vascular, oxidative, inflammatory and metabolic biomarkers that can be measured comparably in animals and humans.
C. Clinical research			
The natural history and recovery trajectory after altitude exposure remain poorly defined.	Define the magnitude, time course and modifiers of reproductive recovery.	Prospective longitudinal cohorts with baseline and exposure sampling plus pragmatic post-return assessments at approximately 1, 3 and 6 months.	WHO-standardized semen parameters, TMSC, SDF and LH/FSH/testosterone; recovery time; altitude dose and major confounders (sleep, exercise, nutrition, cold exposure and psychological stress).
Clinically meaningful exposure thresholds, high-risk groups and effective interventions are not established.	Develop risk stratification and test prevention or recovery strategies.	Adequately powered prospective cohorts followed by controlled intervention trials in defined exposure groups.	Persistent semen abnormalities, time to recovery, SDF/sperm DNA integrity, intervention safety and, where feasible, conception/pregnancy or other fertility-relevant outcomes.

BTB, blood-testis barrier; CASA, computer-assisted sperm analysis; HIF-1α, hypoxia-inducible factor-1 alpha; SDF, sperm DNA fragmentation; SSC, spermatogonial stem cell; TMSC, total motile sperm count.

Fourth, the magnitude, time course and inter-individual variability of reproductive recovery after high-altitude exposure remain poorly defined because baseline-to-return longitudinal cohorts are scarce. A pragmatic longitudinal design could include pre-exposure baseline, sampling during exposure and follow-up at approximately 1, 3 and 6 months after return, while recording altitude, sleep, exercise, energy intake, cold exposure and psychological stress.

Fifth, the independent contribution of post-testicular transport and epididymal maturation defects to semen abnormalities remains uncertain. Segment-resolved animal studies of the rete testis/efferent ductules and epididymal regions should distinguish intratesticular spermatogenic injury from post-testicular defects in barrier function, luminal composition and sperm maturation.

These unresolved questions and corresponding priorities are summarized in **Table 6**, organized separately into basic, translational and clinical research.

## 10 Discussion

High-altitude hypoxia-induced male reproductive dysfunction should not be reduced to direct hypoxia-induced germ-cell apoptosis. Human studies provide the most direct evidence that high-altitude exposure can be associated with changes in semen quality, reproductive hormone profiles and recovery after descent, although the magnitude and reversibility of these changes vary considerably across exposure conditions and populations. Experimental hypobaric-hypoxia and animal studies extend these observations by demonstrating testicular structural injury, oxidative and metabolic stress, germ-cell loss and cell-specific molecular alterations. These two evidence streams are complementary but should not be considered equivalent: human studies define the clinically observed reproductive phenotype, whereas experimental models primarily inform the biological mechanisms that may underlie it.

Against this evidence background, the integrated model proposed here places testicular oxygen homeostasis upstream of local injury. When compensation is exceeded, the interstitial vascular endothelial niche–Leydig–cell–immune axis and the seminiferous Sertoli–cell–BTB–germ–cell support system jointly propagate reproductive injury. Peritubular myoid cells, the basement membrane and ECM connect these compartments as a continuous niche. Systemic HPG–axis regulation may further link whole–body adaptation to local testicular function, whereas sleep, energy balance, post–testicular factors and emerging immune–metabolic signals may modify the magnitude and inter–individual variability of reproductive impairment. Ultimately, whether injury reaches spermatogonia or the SSC niche may determine whether recovery is rapid and complete or delayed and incomplete.

A further value of this framework is that it extends the interpretation of altitude–related reproductive dysfunction from injury alone to recovery biology. Semen abnormalities reflect downstream reproductive output but do not necessarily indicate whether spermatogenic reserve has been preserved. We therefore propose that the depth of injury—whether it remains limited to mature sperm or differentiating germ cells, or extends to spermatogonia and the SSC niche—may contribute to differences in the speed and completeness of recovery after return to normoxia. In this sense, the framework generates three central testable questions: whether a measurable local oxygen threshold separates compensation from decompensation; whether dysfunction propagates between the interstitial and seminiferous compartments; and whether preservation of SSC–niche integrity predicts reproductive recovery. Thus, the proposed framework is intended not simply to rename established mechanisms, but to connect systemic exposure, local compensation, compartmental interaction and spermatogenic reserve within a unified model of injury and reversibility. This framework may support more precise experimental design, risk assessment and follow–up strategies for men exposed to high altitude.
